# Supplementary figures and images for: Sticky Pi is a high-frequency smart trap that enables the study of insect circadian activity under natural conditions
Source: PLoS Biol. 2022 Jul 7;20(7):e3001689. doi: 10.1371/journal.pbio.3001689 (PMC9262196; doi:10.1371/journal.pbio.3001689)

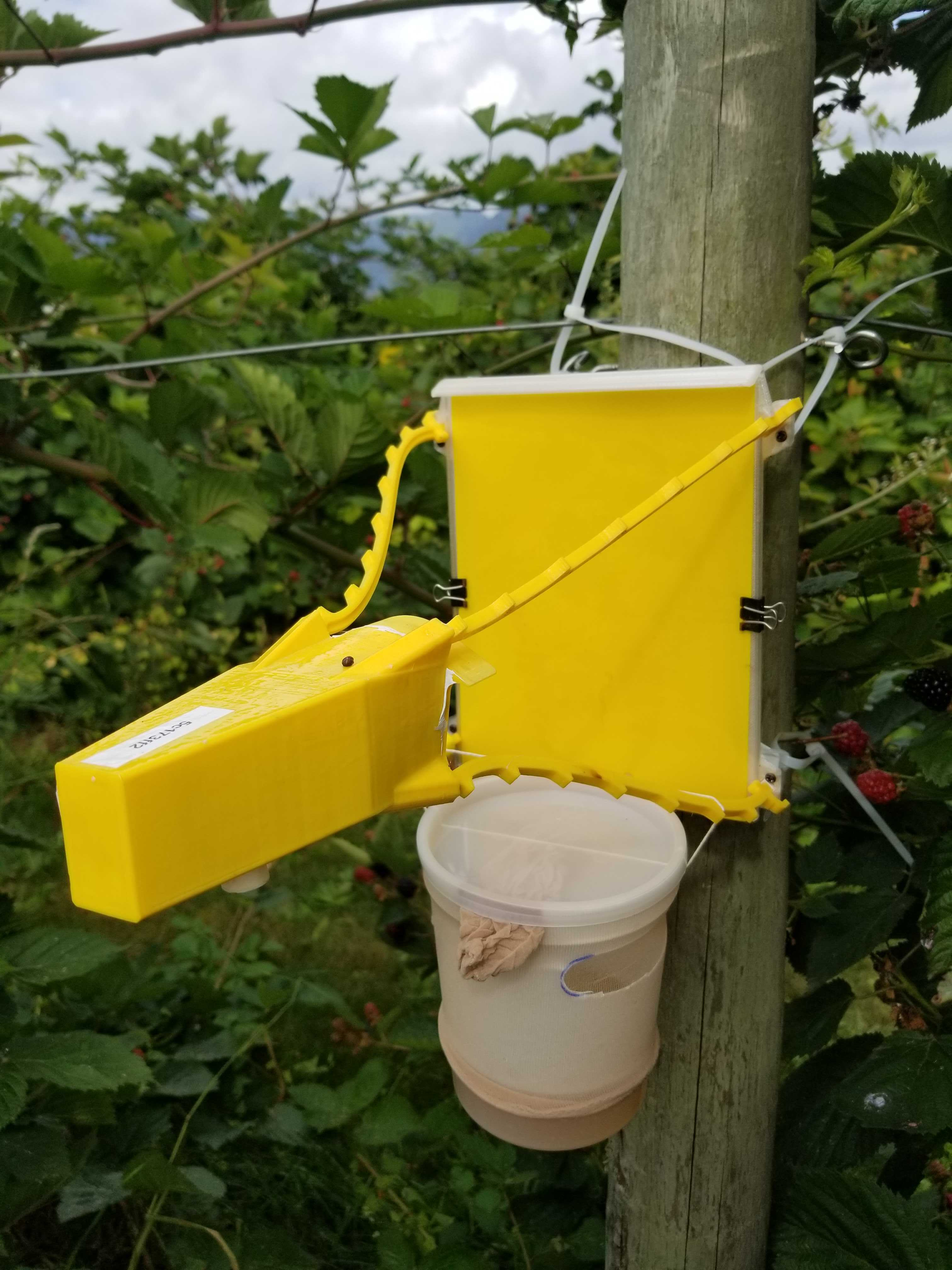

Supplement: S5 Fig — Sticky pi device (top) with an olfactory bait (bottom). The bait consists of a container holding 200 mL of apple cider vinegar protected behind a thin mesh. Apple cider vinegar was replaced weekly during trap maintenance. (TIFF) [file pbio.3001689.s005.tiff]
