# Supplementary material for: Sticky Pi is a high-frequency smart trap that enables the study of insect circadian activity under natural conditions
Source: PLoS Biol. 2022 Jul 7;20(7):e3001689. doi: 10.1371/journal.pbio.3001689 (PMC9262196; doi:10.1371/journal.pbio.3001689)
Supplement: Attachment — Submitted filename: geissmann_et_al_2022_response.pdf [file pbio.3001689.s016.pdf]

THE UNIVERSITY OF BRITISH COLUMBIA

Quentin Geissmann
Dept. of Microbiology & Immunology
BC Canada
V6T 1Z3
+1 236 979 3141
quentin.geissmann@ubc.ca
https://quentin.geissmann.net/

Dear editors of *PLOS Biology*,

April 29, 2022

We would like to thank the editorial team and all three reviewers for their thorough work on our original submission. We believe that the comments and questions raised have considerably helped us improve our manuscript. We are glad to be resubmitting a version with some major revisions that address all the points raised during the review process. In this letter, we first summarise the main changes and, second, provide a point-by-point answer to each of the reviewers' comments.

Summary of changes:

In the original review, the reviewers raised concerns about the performance and applicability of the machine learning tools, in particular from reviewers #1 and #2. In addition, reviewer #2 pointed out that our web-documentation did not explain how to perform the machine learning in practice. Thanks to these comments, we were able to dramatically improve the performance and generality of insect detection and tracking. We did this as follows:

- We annotated and added more than 150 new images, including data kindly provided by the community, to the dataset for our segmentation algorithm. Given that these images were acquired with different optics (some of them with a desktop scanner), we have considerably improved the generalisability of our tool.
- Retraining our algorithm, with a more diverse dataset, has resulted in a massive performance gain on the original data for large insects ($> 2mm^2$): we now have $> 90\%$ precision and recall. In other words, we halved our false-negative rate. We also achieve high performance on the Raspberry

Pi HQ camera, which was released after the original work, and will be the new standard (> 92% recall and > 96% precision on all insects). We also hope to continue collecting community images and improve scope and performance further.

- To improve usability and replicability of the machine learning tools, we now have a dedicated documentation page (https://doc.sticky-pi.com/ml.html) that explains how to install our tools. We packaged python standalone executables to facilitate the partial use of our tools (rather than relying on a heavy API/database), we provide examples of reference annotated data for the users to practice and we describe annotation procedures. We believe this largely extends the scope of our algorithms, making them much more adoptable.
- We have reprocessed the relevant manuscript data and redrawn all figures that depended on the updated machine learning.
- We discussed a number of points including the difference between camera-based and sensor-based options; possible pitfalls such as the saturation of traps by insects, which justified a new supplementary figure; the biases in approximating insect activity from capture rate; and the effect of environmental variables.

Sincerely, On behalf of all authors,

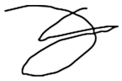

Quentin Geissmann, PhD

Human Frontier Science Program Postdoctoral Fellow
Dept. of Microbiology & Immunology
The University of British Columbia

Point-by-point responses to reviewers

In the text below, we address, point by point, all comments from the three reviewers. For readability, the reviewer's comment is quoted verbatim, in black, and our responses are shown in purple font.

Reviewer #1, Eamonn Keogh: Very nice paper. Well motivated, well explain, great experimental design, high reproducibility, good illustrations, open source etc.

However..

I think this is the wrong solution.

1) You are getting counts every 20 minutes, but that will obscure very finely timed behaviors, consider fig 5 of [a]. It would be easy to get every second.

We thank the reviewer for the positive feedback on the manuscript and helpful suggestions that have improved both presentation, clarity, and rationale for our approach. Our goal was to provide temporal data to ask fundamental questions related to circadian community ecology. In the context of circadian biology and, for instance, including weather effects, we think 20 minutes is the appropriate compromise between temporal resolution and battery/data (we also limit the impact of light pollution, compared to a continuous approach). For instance, even in laboratory setups, circadian data is often aggregated over several minutes for analysis. We now included a discussion on the rationale behind the 20-minutes interval (see lines 271-277 of the version with changes tracked). Our tool could also be modified to answer questions that would require a finer temporal resolution.

2) The proposed system is computationally very heavyweight, but simpler hardware and software can do a better job [a].

Indeed, there are sensor-based (as opposed to camera-based) systems that can perform very well. We now include a discussion of the comparison with such tools. Using images results in expert-understandable results. Namely, insect images can be directly annotated (as experts can visually ID insects, but cannot often do so using acoustics/wingbeat patterns, which is mostly undocumented in entomology). Images also have the advantage of being independent of temperature and humidity (whilst these variables impact wingbeats patterns).
In the work suggested by reviewer #1, the authors have had to capture and keep insects alive to acquire ground truth in the lab – for many species, this can be very limiting. In addition, visual features can provide very valuable unique information. In our manuscript, male D. suzukii (spotted-wing drosophila) have distinctive spots which are crucial for species identification. We now discuss further the strengths and limitations of using images (see lines 278-291 of the version with changes tracked).

3) The system is destructive, you can only count dead insects, but it would be sometimes useful to count live insects, without killing them (for A|B tests etc)

While we acknowledge it can often be useful to count live insects. We also argue it can be advantageous to kill and keep them for, at least, two reasons: 1. We can almost guarantee not capturing twice the same insect, so we reduce re-observation bias. 2. We can keep individual trapped insects for subsequent ID (e.g., using DNA barcoding). Indeed, it is very hard to match data with insects unless they were killed or kept in individual containers. For very diverse groups such as wild insects, taxon identification is not trivial and keeping physical specimens and diagnostic genetic material associated with observations for repeatability and posterity is absolutely critical. Thus, the destructive nature of the sampling is not a limitation, it is a necessity in the context of biodiversity research on insects outdoors, particularly as this tool is meant to be applied in places where insect community composition may be very different and validation of identification will be crucial (see Turney et al., 2015). This is now discussed (see lines 285-290 of the version with changes tracked). Altogether, we argue that sticky cards and timelapse images are preferable in scenarios when little is known about the target insects, or no ground truth data can be acquired a priori. Therefore, our tool can be applicable in naive contexts, with a posteriori labels.

4) At some point you have sticky trap situation, either with dead insects, or with dust. At that point you can no longer say anything. Using [a] means you can always count.

Sticky cards can indeed become very crowded; however, we show that crowding does not interfere with our insect counting and identification approaches, and that, in our context, the capture rate was linear throughout an experimental week, which suggests dust was not an issue either. In our data, replacing cards weekly, we observed no effect of the number of previously trapped insects on the probability of capture of subsequent insects. In our current revision, we included a new supplementary (Supplementary figure 6) figure showing traps are not overcrowded in our context. It is however a valid point that we expect to be sometimes an issue and that should be considered by future users, which we now discuss (see lines 224-234 and 292-296 of the version with changes tracked).

I do worry about the generalizability of deep learning. It is difficult not to have them overfit. How robust are they to type of camera, color of sticky trap, distance from lens to trap, ambient light etc

Thank you. We have addressed this concern by adding new images from different sources to our dataset and retraining our model. This is described in length in the response to reviewer #2.

-
The videos are very helpful.
I hope when deployed, you put a grill of some kind around the trap, to prevent birds and bats being caught in the sticky traps. In some places, you will also need to exclude geckos and lizards (I have found Teflon sheets around the support poles works very well)

We are glad the reviewer found the videos helpful. Thank you for these additional suggestions as these are important considerations in deploying traps in distinct geographical

regions. We originally designed the “legs” of the Sticky Pi to fit a cage against birds, but could not ensure that it would be harmless for birds. With time-lapses, predation is not always a problem as long as insects stay long enough on the card before they are predated. We have now added a sentence to make the reader aware of this possible issue (see lines 70-71 of the version with changes tracked).

[a] Yanping Chen , Adena Why, Gustavo Batista, Agenor Mafra-Neto, Eamonn Keogh. Flying Insect Classification with Inexpensive Sensors. Journal of Insect Behavior 2014 http://alumni.cs.ucr.edu/~ychen053/InsectBehaviour_059.pdf

Reviewer #2: In this methods article, Geissmann and colleagues present a novel tool to perform insect surveys in the field. The Sticky Pi tool consists of one (or more) 'recorder' devices which consist of a camera, weather station and a sticky insect trap. The trap is photographed at a set time interval. The data is then periodically transferred to a server using a 'data harvester', another device that prospective users need to build.
To analyze the large amount of data the authors employ a three step analysis: first animals found on the trap are separated from the background, then individuals are tracked over time and finally each individual is assigned to a species. The authors collect and analyze a total of 3 datasets to support the functionality and usefulness of the tool. The results of the analysis are as expected (but appropriate statistics needs to be added, see below) indicating that Sticky Pi performs as claimed.
This manuscript is submitted as a methods paper. As such the reader should expect that enough information is provided to build the setup and run experiments as described in the manuscript. The paper is accompanied with a beautiful website which explains how one can build the hardware components. However, the generalizability of the machine learning based analysis pipeline is far less clear.
Overall, the authors present an important and exciting tool to automate insect surveys, but the manuscript would greatly benefit from additional information as well as clarifications related to the analysis pipeline which is an integral part of the Sticky Pi tool. Moreover, it would be important to further benchmark the performances of the species identification (see below).

We thank the reviewer for the positive feedback and thoughtful critique. In particular, we feel the reviewer’s questions and suggestions have helped with the clarity of the presentation, and we hope will facilitate others using Sticky Pi. We have thoroughly responded to the comments and concerns below and believe our manuscript is much improved as a consequence.

Major concerns:

A main concern with the information described in the manuscript is the applicability of the analysis pipeline by other users. Could the authors clarify the following points?

1) Can the Universal Insect Detector be used with different datasets?

Yes, the Universal Insect Detector can be used with diverse datasets. As part of the revisions for this manuscript, we extended the scope of the UID. We added to our dataset new images from other authors (N= 31), including different cameras or scanners, as well as images using another camera/lens (N=140) (acquired by ourselves in 2021). Training the UID on the whole dataset (original and foreign images) considerably improved performance (see below). Rather than retraining the UID on new data only (which should take a lot fewer images), we recommend building a community (universal) dataset for this generic task. We hope the publication of this method will spark such endeavour and help us to source more images to increase the scope even further.

If yes, did you use the identical network for the 3 datasets presented in the manuscript?

The same models were used for the UID of all field data. For the lab dataset, we used an initial version of Mask-RCNN, which we kept separate. This is because laboratory images were acquired with IR cameras that have overall poor optics (which was satisfactory in our case since all insects were the same species and tracking was not needed). We explain this more explicitly in the paper (see lines 155-161 of the version with changes tracked).

If you have other data that can not be used with the Universal Insect Detector, how would you go about training the network?

As part of the current revision we 1., developed standalone python tools to perform ML tasks, including UID (that can now be done on its own, without having to install database/server…), and 2., extensively documented how to use our ML tools (https://doc.sticky-pi.com/ml.html). We have included information on installation, use and annotation of the data. We also now provide toy data for users to practice.

2) Can the Siamese Insect Matcher be used with different datasets than those presented in the manuscript?

The siamese insect matcher can, in principle, be used on other data, but it is specifically designed to handle time-lapses, which are still uncommon data. In this revised manuscript, we generalised the SIM by training it on both the original and new (2021) data, with a different camera sensor/lens. It should be noted that our model explicitly uses distance moved and area of the insect to compute similarity. We expect the SIM to be rather robust to new images as Siamese networks are designed to abstract the similarity between images (rather than label individual images). Furthermore, distance moved and area are not visual properties per se, which makes them robust to image quality. Since both UID and SIM were retrained with new data, the dependent figures 5, figure 6, and supplementary figures 2 and 4 were also rebuilt. The statistics for these figures, in the text, were also updated, this resulted in no qualitative difference.

Did you use the identical network for the 3 datasets in the manuscripts?

Only for the field data. As explained above and, now more clearly in the manuscript, we did not track insects in the lab experiments – since images had consistent IR lighting and individual insects are all of the same species, we could infer the numbers accurately by

‘simply’ counting segmented instances on each frame. We have clarified this in the current version, (see lines 155-161 of the version with changes tracked).

If not, how you would go about training the network with new data?

The SIM is now also documented. Toy data and annotation procedures are provided.

3) Obviously, the Insect Tuboid Classifier needs to be adapted to different data. Is it realistic to expect to just re-train the network with one's own classifiers? Or do you foresee the need to adapt the ResNet50 architecture itself?

It is realistic to retrain the networks with one's own data. If the images are clear and numerous, we anticipate good performance. We do not anticipate it would be necessary to change the network architecture.

Please clearly outline steps someone would need to take to get optimal results.

Optimal results rely greatly on the quality and size of the training data. This process may be time-consuming and require expert knowledge. Furthermore, models will be context-dependent (season, location, ...), which will require significant annotation efforts. To address this issue, we released our annotation tool as a collaborative standalone Rshiny app (packaged as a docker service), which can be deployed on local or remote machines. This means multiple experts can work together and non-experts can use weblinks to pre-screen insects and transfer annotations to the appropriate experts. We also documented how to deploy and use this tool and provided documentation on our website.

4) For each of the networks: How does one go about labelling the data wherever it is necessary? Do you provide an interface to perform labelling or do you recommend other software?

Thank you for this feedback. We realise that the original version did not describe how to use the machine learning tools from a practitioner's perspective. As described in the previous responses, we now have a comprehensive tutorial and more accessible tools as well as a description of the annotation process and toy data for the three models (see https://doc.sticky-pi.com/ml.html).

Please make sure the labelled data can be feed into your analysis pipeline without problems. The authors need to show that their analysis pipeline will be accessible and useable for other researchers.

Yes, this is true. Thanks for this feedback. The updated documentation and toy data should considerably help adoption and contributions (see https://doc.sticky-pi.com/ml.html).

Another concern is the sensitivity of the species identification algorithm. The recall rate reported in Supp. Fig . S2 is 70% when all objects were included — this rate is modest. An increase to 80% is obtained when the smallest insects were excluded from the analysis. While the size filter might be unavoidable, the sensitivity of a 80% recall rate suggests that

the abundance of some species might be underestimated. For this reason, it would be important to benchmark the performance of the classifiers in lab conditions. For instance, the authors could prepare a mixed population of D. melanogaster, D. suzukii and a handful of other insects. This population could be placed in a cage with a Sticky Pi device (as was done for Figure 4). In these controlled conditions with known species, the authors should be able to assess the performances of the automated identification. This analysis would be useful to validate the results of the tracking of D. suzukii versus other
Drosophilidae presented in Figure 5.

Indeed the original performance of the UID was "modest". As part of the revisions, we retrained the model including external data (see above). In addition, we improved the algorithm through data augmentation and by accounting for insects that are on the edge of the image – as opposed to the edge of a sub-image (i.e., a tile), (see lines 378-384 of the version with changes tracked). Altogether, we dramatically improved performance on the original data, the updated version has now an 80% recall on all objects and 90% when small insects are removed, which is half the error. In the original dataset, the image quality is still limiting, and small insects appear intrinsically difficult to classify. Using new optics and similar conditions (the 2021 dataset), we have more than 90% recall and precision. We will document how to assemble this improved Sticky Pi version in the next few months. Altogether, we think these software and preliminary hardware improvements address this concern. It is worth noticing that the recall of actual insects through time is likely higher than the recall on single frames. Indeed, the SIM reconstructs insect trajectories even when insects are missed over several frames. Although the actual recall is hard to quantify, as the probabilities of detection of the same insect between two frames are not independent, tracking does address some of the recall limitations.

We also believe that laboratory experiments would not address this question very well. Indeed, we consider that UID performance is mostly impacted by variable environmental conditions (light, fog, rain, predators, ...). For instance, in the field, it is usual to observe only partial insects (that have degraded), which is an inherently difficult problem.

Figure 4:
The authors use the data in this figure to conclude that their tool can capture circadian rhythm. Although the data look promising, it is necessary to add appropriate statistics to support statements made in the text.

Thanks, yes. We are now statistically testing for rhythmicity, using t-tests on autocorrelation coefficients, with a 24h lag. In this amended version, we show that the capture rate of LD and DD, but not LL populations, have a high and significant autocorrelation at 24h (see lines 165-168 and 171-175 of the version with changes tracked).

This should also address why the LD control in C and D are different (by eye): You have more captures in D both in LD and DD and a generally lower baseline of morning capture in C and no capture in LL.

We performed pairs of experiments (LD vs DD or LD vs LL), which we replicated over time. In this context, the animals (e.g., different generations) and subtle experimental conditions (e.g. vibrations and air pressure) may differ from week to week, so, unsurprisingly, the data

between the two LD controls slightly differ (although it is qualitatively similar). We have now explicitly pointed out that both DD and LL conditions should be compared with their internal LD controls only – and not between experiments (see lines 154-155 of the version with changes tracked).

Why did you remove high frequency noise in this dataset but not the others? (Line 449)

We now describe how the processing of our laboratory data (IR, Drosophila melanogaster only) differs and why we applied a simple filtering approach as opposed to the tracking (see lines 154-161 of the version with changes tracked). In short, tracking is overkill (and possibly less accurate) if we know that all individuals are the same species, the image quality is consistent and there is no occlusion. Likewise, the classification is already done at the segmentation stage since we only have one class (Drosophila melanogaster vs background).

Figure 5: In this well-designed figure, the authors compare male D. suzukii capture with wasp capture. They conclude that: "Our results corroborate a distinctive crepuscular activity pattern for male D. suzukii and other putative drosophilids. In contrast, Figitidae wasps were exclusively diurnal." Please provide appropriate statistics to support this statement.

We now provide some statistical support (bootstrap confidence intervals). It is difficult to show statistically that an animal is "crepuscular". Therefore, we compared our observations to plausible null hypotheses where insects would have a time-uniform probability of capture (see lines 192-196 of the version with changes tracked).

In addition, the authors state that baiting increases the number of male of D. suzukii being captured. In addition to the stastistics, please provide an appropriate plot showing these results.

Unless we misunderstood reviewer #2's comment, we think the colours in figure 5 show clearly, together with the statistics in the text, that bait (blue) has a very large effect on drosophilid capture rates, vs unbaited (red). Does reviewer #2 suggest we plot an aggregate value rather than a time series?

Minor issues:
General:
What are the advantages of using white light for illumination? You showed in Figure 4 that IR light can be used. Please discuss this point.

Yes, using IR cameras means we reduce the quality of images. Most optics are designed for visible lights (IR filters are built-in). Adding IR (in addition to visible light) causes chromatic aberrations and is particularly difficult in the field, in the presence of sunlight. Using IR-only would also mean we lose colour contrast – and likely decrease identification performance. Without going into too much detail on the optics, we explain this in the revised manuscript (see lines 73-77 of the version with changes tracked). In practical terms, it is also more affordable and easier to debug visible light setups (white light) – for instance, one cannot tell by eye if an IR light is failing during assembly.

Website:
Please update the BOM: for example the link for the GPS module says 'currently unavailable' which would make it impossible to build the data harvester.

Thanks, done.

Link to 3D printer files doesn't work as expected: Using the link to Onshape, the reviewer only gets one file 6-Pin Shrouded Header (IDC) Right Angle. Link to OS for data harvester doesn't work.

Thanks for such a thorough review and for verifying the validity of the additional web material. These two points are now addressed.

Figure 1:
Please clarify whether the sticky pad it must be transparent since the LEDs placed behind the pad? Are all sticky pads/cards transparent or did you have to select a particular brand?

All sticky cards we tested are sufficiently translucent to allow enough light through. One of the advantages of using white light is that it is also robust to different absorption spectra (i.e., trap colours). We have added a comment about that as well (see line 73 of the version with changes tracked).

Consider adding a picture or illustration of the data harvester.

The documentation now has a picture of the data harvester (https://doc.sticky-pi.com/hardware.html#data-harvester).

Figure 3:
A) Please clearly indicate that input is from Siamese network (not images).

Thanks, this is now clearer in the figure legend.

B) Please consider using a heatmap as the presentation representation is very hard to read.

Thanks, we now have the confusion matrix as a supplementary table. We could also consider adding a heatmap version in the main figure.

C) In legend, F1 score is not explained.

Thanks, f1-score is now explained in the figure legend

D) Please consider adding scalebars - if the reader is not mistaken, everything has been rescaled. Therefore, out-of-focus images (e.g., 2) are of insects which are smaller than 'sharper' (e.g. 7) ones? This could be made more explicit in the legend.

Thanks, We have added scale bars. Note that we had not kept the original descriptors for each tuboid used as a visual example. Therefore, we selected new representative images

(from which we had and kept the original scale), which is why the 18 tuboid images are different.

Figure 4:
Please rearrange the legend (LD, LL and DD at top): Currently the LL is in the center while all data is presented in a left (LD/LL) and right (LD and DD) column.

Thanks, this is now fixed.

If the reader understands correctly, y-axis in 4C and D is the same as in Fig5 A/B and Fig 6A. Please label consistently throughout the manuscript.

Thanks, this is now fixed.

As much as possible, please normalize the size of indivudal figures. At the moment, Figure 4 seems to be shrunk compared to Figure 5. This is obvious when comparing the labels of the plots.

Thanks, this is now fixed.

Figure 6:
Missing x axis label under 'Culicidae'.

Thanks, this is now fixed.

Figure S3:
Regarding the calculation of M(m,n): In methods (line 275), you write that "Given a pair of objects m, n, in images Xi and Xj, we have the binary masks Am and An of m and n, respectively. We then use the same function D to compute two similarity values S(m, n) and Q(m, n)." In your Figure S3A you have a total of 3 inputs with the lower looking like the difference between the binary mask of the two? Please elaborate in text why you chose to show the 3 inputs. Following the Siamese network you use a "custom, four-layers, fully connected neural network" (line 275). Why did you choose that particular architecture and those inputs? Do you expect this network to perform well on different datasets?

Thanks, we now added our reasoning behind this architecture (see lines 404-414 of the version with changes tracked). Briefly, S captures whether two insects, in two consecutive frames, look the same. Q, on the other hand, looks in the second image where the first insect would be, if it had not moved, and compare it to the first insect image. If the original insect has not moved, then Q is high. Just using S, the naive similarity, means we cannot always tell if an insect has moved to a close location or if a new, similar insect appeared nearby. This is particularly important if an insect is missed (segmentation false negative) in the second frame. In this case, Q would be high. These considerations add a certain level of non-linearity in computing M (e.g., if Q is high AND S is low OR the distance moved is small, then …). To account for these non-linear relationships, we used a fully connected four-layer network, which was sufficient to account for non-linear behaviours whilst keeping the number of parameters low (compared to the rest of the network).

Discussion:
It would seem appropriate to discuss the different approach Sticky Pi takes (decentralization of computation) compared to other published tools like this https://doi.org/10.3390/s21020343.

Thanks, we have added a statement that explains how our decentralised solution helps to scale to the landscape level (see lines 249-254 of the version with changes tracked).

Cover page: There appears to be typos in affiliation #3.

Unless we are mistaken, this comment seems to refer to the word “xʷməθkʷəy̓əm” (Musqueam). The campus of the University of British Columbia is located on unceded land. We often use the North American phonetic alphabet to represent indigenous names. Hopefully, this will be approved/addressed at later editorial stages.

Reviewer #3: The authors present a 'High-frequency monitoring tool to study insect circadian biology in the field' with applications in 'biodiversity monitoring, pest control, phenology, behavioural ecology, ecophysiology'. Their methodology represents an interesting advance in temporal monitoring of insects (and other captured species) in the field. The new methodology is tested in laboratory and field conditions in both baited and unbaited scenarios. While the computational limitations of the methodology receive scrutiny and validation, the manuscript is less sophisticated in discussing potential limitations in the interpretation of the collected data. The temperature and humidity conditions in the field were presumably monitored by the devices, but they were not reported as far as I can tell. This is relevant as diurnal insect behaviour changes strongly with environmental temperature patterns.

We thank the reviewer for their valuable feedback and suggestions that have helped improve our manuscript. We now report temperature and humidity patterns and discuss their importance (see lines 333-342 and 521-522 of the version with changes tracked, and the new supplementary figure 7).
Regarding field experiments, we do not claim that the observed behaviours are either purely circadian or solely responses to the environment. Likely, the two components play a role. Disentangling the respective effects of time of the day and temperature (or humidity), the latter being highly explained by the former, on capture rate is beyond the scope of this method-description paper. We performed our field experiment on two small homogenous plots, humidity and temperature hardly varied between devices, which makes such a study statistically difficult to address. This question will be much more interesting at the landscape level, which is the topic of some of our future studies.

Formally, what is measured is not the temporal field activity of insects per se, but rather the pattern of their trapping on the sticky surface of the device used with or without a bait being present. This adds further limitations that are worth acknowledging explicitly in addition to the limited taxonomic resolution that was mentioned. Trapping patterns will depend on the presence of bait (see notable difference for D. suzukii), competition with attractants present in the field, and further interactions with biotic and abiotic factors.

Yes, this is a very valid point. We now acknowledge in the text that what we record is indeed not the activity, but the capture rate. This is a limitation in some cases. As reviewer #3 points out, capture may be influenced by many factors that change throughout the day and season. We now discuss this point (see lines 312-327 of the version with changes tracked). We also think this property could be very helpful to study the behavioural ecology of preference and foraging. For instance, experimenters can use pairs of traps that contrast for bait chemistry or colour and compare the timing of capture rates. This setup could help ecologists understand how insects are attracted by different resources at different times of the day, or in a weather-dependent fashion. This point is now also discussed in a dedicated paragraph (see lines 327-332 of the version with changes tracked).

In the case of the 'Sticky Pi' device one would also want to explore possible unintended impacts of light flashes associated with image capture as well as the impact of prior captures on the trap on subsequent ones in association with emitted attractant/repellent signals.

Given that light flashes only occur once every twenty minutes, and are only two sub-second (circa 100ms) pulses, we hypothesise they are unlikely to largely impact capture rates. We have included a statement about how using 20min intervals reduces light pollution compared to more continuous options (see lines 276-277 of the version with changes tracked). We also now acknowledge the possibility the trapped insects could impact the probability of subsequent captures. We investigate this possibility for the second field experiment with a new supplementary figure (supplementary figure 6). In our context, we did not find any effect of the number of trapped insects on the capture rate of specific taxa.

It is also not clear whether the trap in its current form was comparatively tested against any other designs. A side-by-side validation relative to alternative trapping methods in the same field conditions would provide much clearer insight into 'Sticky Pi''s usefulness in a particular context than the notion that D. suzukii male behaviour appeared to be 'crepuscular' under field conditions with unspecified environmental profiles.

The very reason we designed Sticky Pi is that there was no viable alternative to automatically record the capture rate over time in this context. Therefore, it is inherently difficult to benchmark our tool against other automatic methods. For instance, for the well-studied pest D. suzukii, a recent study relied on a manual sampling of traps that aggregated insects over two-hour time windows (Swoboda-Bhattarai and Burrack, 2020), which limits the resolution and scale of this time of studies.
We hope the openness of our design will make it simple for other authors to compare alternative methods against Sticky Pi.

In summary, while this study should be welcomed as a technical advance that may have a number of useful applications, it lacks rigorous field-based validation as well as a discussion of potential pitfalls associated with linking recorded data to field behaviours.

Thanks to the reviewer's feedback, the manuscript is now much more rigorous and discusses the pitfalls and limitations that were pointed out.
