# Supplementary material for: Sticky Pi is a high-frequency smart trap that enables the study of insect circadian activity under natural conditions
Source: PLoS Biol. 2022 Jul 7;20(7):e3001689. doi: 10.1371/journal.pbio.3001689 (PMC9262196; doi:10.1371/journal.pbio.3001689)
Supplement: Attachment — Submitted filename: geissmann_et_al_2022_response_to_minor_corrections.pdf [file pbio.3001689.s017.pdf]

Dear Editor of *PLoS Biology*,

We would like, once again, to thank the editorial team for their thorough work on our revised submission. We have addressed all minor comments raised by the editor in the present resubmission.

In the text below, we address, point by point, all comments from the editor. For readability, the editor's comments are quoted verbatim, in black, and our responses are shown in purple font.

Editor:

1) After some discussion within the team, we think the title should be edited for clarity. If you agree, we suggest it be changed to something like "Sticky Pi is a high-frequency smart trap that enables study of insect circadian activity under natural conditions"

*Thanks for your suggestion. We are pleased with the title suggesting the traps can do other things. However, we feel it should be "Sticky Pi is a high-frequency smart trap that enables **the** study of insect circadian activity under natural conditions". We have updated the title in the manuscript and submission form accordingly.*

2) Thank you for providing supplementary data tables and videos on Figshare. In order to be compliant with our Data Policy, which requires that all data be made available without restriction, we request that you also provide, as a supplementary file or deposition in a publicly available repository, the underlying data used to generate each figure.
Note that we do not require all raw data. Rather, we ask that all individual quantitative observations that underlie the data summarized in the figures and results of your paper be made available.
Please ensure that you provide the individual numerical values that underlie the summary data displayed in the following figure panels as they are essential for readers to assess your analysis and to reproduce it:
Fig 4A-F; 5A-B; 6A-B; Fig S2 D-E; Fig S4

NOTE: the numerical data provided should include all replicates AND the way in which the plotted mean and errors were derived (it should not present only the mean/average values).

**Please also ensure that figure legends in your manuscript include information on where the underlying data can be found, and ensure your supplemental data file/s has a legend.

**Please ensure that your Data Statement in the submission system accurately describes where your data can be found.

For more details on our data availability policy, see here:
http://journals.plos.org/plosbiology/s/data-availability

Thanks, the data for all requested figures were uploaded on figshare (10.6084/m9.figshare.19764199.v1), in a single entry and all the data and metadata fields

have been described. This reference was added to the bibliography and referred to in the relevant figure legends.

3) In order to comply with PLOS' ethics policy regarding field and observational studies (https://journals.plos.org/plosone/s/submission-guidelines#loc-observational-and-field-studies), we request that you add the following information to your methods section:

-Please provide details on permits and approvals obtained for the field experiments performed here, including the full name of the authority that approved the study and the approval numbers; if none were required, please explain why

No permits or approvals were required for field experiments performed here, as they did not involve human or animal subjects, and were performed on the grounds of the research station (the Agassiz Research and Development Centre, Agriculture and Agri-Food Canada) where the second author is employed.

- Please indicate whether the land accessed is privately owned or protected

The land is owned by the research institution employing the second author and is not protected.

- Please indicate whether any protected species were sampled

Based on the identifications we performed on our samples, to our knowledge, no protected species were sampled.

4) Please provide scale bars for images in Figure 2 and Figure S2.

Thanks for pointing this out. We added scale bars on both figures.

In addition to these requests, we have deposited our DNA sequence on Genbank (SUB11480448), and referenced them accordingly.

Sincerely, On behalf of all authors,
Quentin Geissmann, PhD

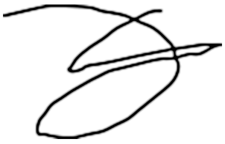

Human Frontier Science Program Postdoctoral Fellow
Dept. of Microbiology & Immunology
The University of British Columbia
